# Management of sphincter insufficiency in patients with neurogenic bladder and bladder augmentation

**DOI:** 10.1007/s00383-023-05506-x

**Published:** 2023-06-28

**Authors:** Seppo Taskinen, Eija Mäkelä, Niklas Pakkasjärvi

**Affiliations:** Section of Pediatric Urology, New Children’s Hospital, Helsinki University Hospital and University of Helsinki, Helsinki, Finland

**Keywords:** Bladder augmentation, Sphincter insufficiency, Bladder neck injection, Fascial sling

## Abstract

**Purpose:**

To investigate the need and efficacy of treatment of bladder neck procedures in patients with neurogenic bladder and augmentation.

**Methods:**

The hospital database was reviewed for patients undergoing enterocystoplasty because of neurogenic bladder during 1990–2019. Diagnoses of patients as well as frequency, type, and efficacy of treatment of sphincter insufficiency were evaluated.

**Results:**

Thirty-seven of 87 patients (43%) underwent surgery because of sphincter insufficiency. The median age at bladder augmentation was 11.9 years (IQR 8.5–14.8), and at the last control, 21.8 years (IQR 18.9–31.1). Bladder neck injections (BNI) were performed for 28 patients, fascial sling operation for 14 patients, and bladder neck closure (BNC) was done for five females. Full continence was achieved in 10/28 (36%) patients with one or repeat BNIs and 9/14 (64%) with sling operation. The outcome of BNIs and sling operations was similar in both sexes. All five female patients with BNC became continent. At the end of follow-up, 64 (74%) patients were dry, 19 (22%) had occasional incontinence episodes, and 4 (5%) had daily incontinence episodes necessitating pads.

**Conclusions:**

Treatment of sphincter insufficiency is challenging in patients with bladder augmentation and neurogenic disease. Only 74% of our patients became fully continent despite treatments for sphincter insufficiency.

## Introduction

Urinary incontinence increases morbidity in patients with neurogenic bladder and presents a significant problem for many patients. Most patients start anticholinergic medications and a clean intermittent catheterization program (CIC) in early life [1]. Although medical treatment can be potentiated by Botox injections [1], for some patients, medical treatment is insufficient to control an overactive or poorly compliant bladder. These patients may undergo bladder augmentation, effectively creating a sufficient volume reservoir with low pressure. Unfortunately, 57.6% of children undergoing bladder augmentation because of the neurogenic bladder also have incompetent sphincters necessitating bladder neck procedures (BNPs) to cure urinary incontinence [2]. Treatment of sphincter insufficiency is not as straightforward as treatment of a high-pressure bladder. Many treatment options exist, including bladder neck injections (BNIs), bladder neck reconstructions, slings, artificial urinary sphincters, and bladder neck closures with variable efficiency and variably laborious surgery [3, 4]. To that end, we initiated this retrospective study to further delineate BNPs in bladder-augmented patients. Our primary objective was to assess the requirement for BNPs and the efficacy of various procedures in this patient group. Our secondary objectives were to evaluate if the requirement for BNPs was linked to specific diagnoses and if the patient’s sex impacted the outcome.

## Methods

With permission from the institutional review board, we reviewed the hospital pediatric urology diagnosis registry to identify all patients with enterocystoplasty. We identified 92 patients who had undergone enterocystoplasty because of neurogenic bladder from 1990 to 2019 in our pediatric hospital. Information on additional surgical procedures, such as bladder neck injections (BNIs), sling operations, or bladder neck closures (BNCs), was sought from the patient files. We included 87 patients with at least 1 year of follow-up information regarding urinary continence. Thirty-seven patients (43%) underwent BNPs because of sphincter insufficiency either before, during, and/or after augmentation (17 (46) % were females). All patients underwent urodynamic evaluation prior to any BNP. Usually, transurethral catheters were used, and BNP was considered in patients with incontinence if they had incontinence episodes despite sufficient bladder volume for age and low maximal detrusor pressure (< 20cmH_2_O) or if their abdominal leak point pressure was less than 20–40 cmH2O. All patients were evaluated with urinary tract ultrasound, function tests, and cystoscopy before surgery. Also, voiding cystography was performed unless the patient had total incontinence. Renal insufficiency or kidney transplantation was not considered contraindication for augmentation.

BNIs were performed as described previously [5]. The youngest patient to undergo the first BNI was 4-year-old, while for the other patients, the procedure was performed earliest at the age of 6 years. The youngest patient to undergo a sling operation was a 6-year-old boy, while the youngest girl was ten. In the sling operation, we harvested a 1.5 cm wide and 15–20 cm long flap of rectus fascia that was left attached in the inferior part. The fascia was turned 1.5 times around the proximal urethra, approached by a vaginal incision in girls and a perineal incision in boys. The free end was sutured to the rectus insertion in pubes. The proper location of the sling was ascertained with the aid of cystoscopy, and competency was tested by manually compressing the full bladder during the operation. When closing the bladder neck or urethra, we now interpose greater omentum or other proper local tissue between the bladder neck and urethra.

### Statistical analysis

In the statistical evaluation, continuous variables are expressed as medians and interquartile ranges (IQR) or ranges. Only categorial variables were compared between the groups using Fisher’s exact test; for the analyses, Statview^®^ 5.0.1, (SAS Institute Inc.) was used. A *p*-value < 0.05 was considered significant.

## Results

**Fig. 1:**
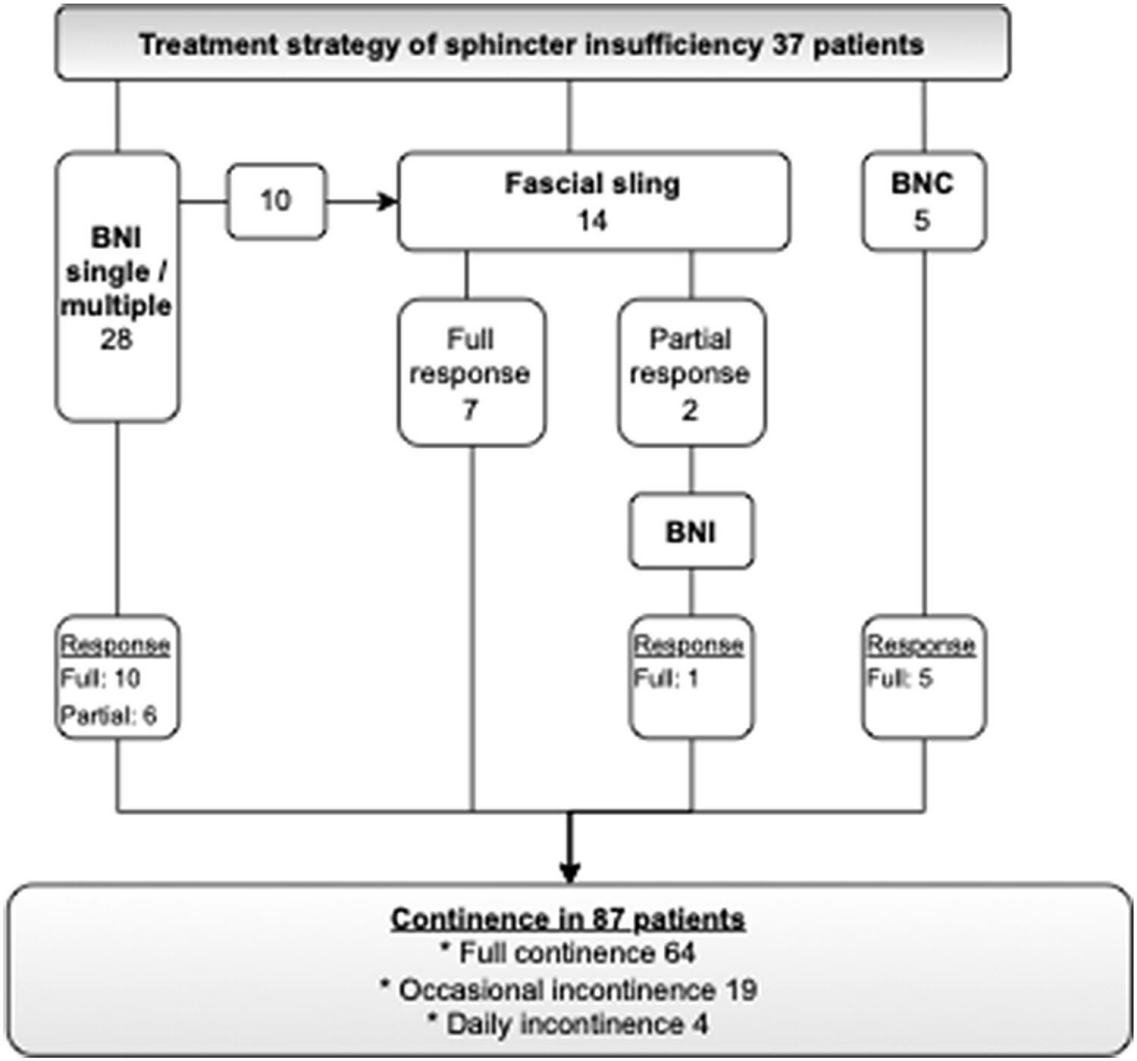
Flowchart of treatment pathways for bladder neck insufficiency in children with bladder augmentation due to neurogenic bladder

As primary outcome measures, thirty-eight of 87 augmented patients (44%) had sphincter insufficiency in urodynamic testing, and 37 patients (43%) underwent bladder neck procedures (BNPs). Thirteen (15%) patients had a continent stoma. The median age at bladder augmentation in those with BNP was 11.9 years (IQR 8.5–14.8), and the age at the last control was 21.8 years (IQR 18.9–31.1). The follow-up time was 10.7 years (IQR 6.3–18.2). In 17 cases, BNP (16 BNIs and 3 slings) was performed before bladder augmentation while the patients were on anticholinergic medication. In 14 cases, BNP was performed at the time of augmentation, and four had also had previous BNP. In 18 cases, BNP was performed after bladder augmentation, and eight had also undergone previous BNP. BNIs were performed for 28 patients (10/17 females and 18/20 males), fascial sling operation for 14 patients (5/17 females, and 9/20 males), and BNC was done for five female patients (Fig. 1).

Ten of 28 patients (36%) receiving BNIs became fully continent (4/17 females and 6/20 male, *p* = 0.72). Three patients became continent with a single injection (one female and 2 males), and seven more with repeated injections. The number of BNIs for a patient was a median of 2 injections (range 1–7). None of the five patients with more than three BNIs became fully continent with that treatment modality only. Sixteen patients had undergone BNI before bladder augmentation.

The sling operation produced full continence in 8/14 (57%) patients (in 2/5 females and in 6/9 males, *p* > 0.9). Of the sling operations, three were performed before, seven were performed at the time of, and four were performed after bladder augmentation. Ten patients had received unsuccessful BNIs before the sling operation, and two patients got BNI after the sling. Other of them became continent. Six of the sling patients had continent stoma operation (one before the sling and five simultaneously with the sling).

Five females underwent bladder neck closure at the time of augmentation. Two of them had caudal regression syndrome with very short urethras, two had traumatic paraplegia (the other had also vesicovaginal fistula) and one had myelomeningocele with very wide bladder neck. All five female patients became continent.

Nine of 17 patients with pre-augmentation BNP also ended to later BNPs because of insufficient outcomes. Five of the remaining eight became fully continent after augmentation (two with sling and three with BNI only). The remaining three had occasional incontinence episodes. Three of 14 patients with BNP at the augmentation also underwent later BNPs. Ten of the remaining 11 became fully continent, and one had occasional incontinence episodes. Eight of the 18 with post-augmentation BNPs became fully continent. Six had occasional, and four had daily incontinence episodes.

At the end of follow-up, 64 (74%) patients with bladder augmentation were fully continent, 19 (22%) had occasional incontinence episodes, and 4 (5%) had daily incontinence episodes necessitating pads (Table 1). In all patients, the bladder had sufficient volume for age with low pressure in urodynamic tests. The reason for incontinence is mainly an incompetent sphincter, but in some cases, perhaps not full compliance with the scheduled CIC program. All patients also had normal s-creatinine levels without hydro-nephrosis. For secondary outcome measures, myelomeningocele was the principal diagnosis in this cohort. Due to the limited numbers of all other diagnoses, we could not evaluate whether there were any differences in the results among the different diagnostic groups.

**Table 1:** Type of procedures because of sphincter insufficiency in 87 patients with neurogenic bladder and bladder augmentation

Diagnosis	*n*	BNI	Sling	BNC	Any surgery for sphincter insufficiency	Fully continent	Occasional incontinence	Daily incontinence
Myelomeningocele	66	26	11	1	28 (42%)	51 (77%)	10 (15%)	5 (8%)
Spinal cord injury and paraplegia or tetraplegia	6	1	1	2	3 (50%)	4	2	
Caudal regression	5		1	2	3 (60%)	3	2	
Lipomyelomeningocele	3		1		1 (33%)	2	1	
Sacrococcygeal teratoma	2	1			1 (50%)	1	1	
Syndromic	2					2		
Meningocele	1					1		
Diastematomyelia	1					1		
Tethered cord	1					1		
All patients	87	28	14	5	37 (43%)	66 (76%)	16 (18%)	5 (6%)

*BNI* bladder neck injection, *BNC* bladder neck closure

## Discussion

Bladder augmentation lowers upper urinary tract pressure and improves urinary continence in patients with neurogenic bladder. We show that while bladder augmentation provides a reservoir of sufficient volume and low pressure, 44% of our patients had concomitant sphincter insufficiency, manifested as incontinence. In the 1990s, when BNIs were a new treatment modality in our hospital, multiple BNIs were sometimes done. Nowadays, our treatment strategy for sphincter insufficiency starts with bladder neck injections up to three times, given that the patient does not present with very difficult incontinence or a very wide bladder neck, and the first injection gives some response or the patients is very young. The patients are offered a sling procedure if BNI is insufficient, or the bladder neck is very wide, or the incontinence is very difficult. We attempt to avoid BNC whenever possible. In this series, we did not perform bladder neck reconstructions. We could not detect differences in the necessity of BNPs among patients with different diagnoses or sex; however, diagnoses other than myelomeningocele were too rare, hindering statistical analysis. With our strategy to treat incontinence due to sphincter incompetency, partial or total continence was reached in 95% of patients. However, only 74% of all our patients and 62% of those who underwent bladder neck BNPs were fully continent at the last control. Six percent had daily incontinence episodes even though the patients had sufficient volume and low-pressure bladders in urodynamic tests, and the sphincter insufficiency was attempted to treat with various methods.

The definition of sphincter insufficiency is not unequivocal in patients with neurogenic bladder, and in many studies, a definition is not given. In the study from Copenhagen, 35% of 80 patients with myelomeningocele were considered to have incompetent urethral function [6]. However, in a large study with 413 augmented spina bifida patients from Indianapolis, 57.6% had undergone BNPs [2]. In another study from Miami, 42.9% of 147 augmented patients also underwent sling surgery because of incontinence [7], convergent with our experience, with 43% of the augmented patients undergoing BNPs because of incompetent sphincters.

Although BNI is the least efficient BNP for incontinence, we have selected it as a first-line treatment in most cases unless a fully incompetent bladder neck since it is a mini-invasive day surgery procedure and suitable even for younger patients. In previous studies, the efficiency of BNIs varied, producing full continence alone or as an adjunct to other BNPs in 7–40% of patients (33% [8], 25% [9], 40% [10], 35% [11], 19% [12], and 7% [13]). Apparently, the variation in the results depends on many factors, including patient selection and the definition of continence. In our hands, a single BNI produced full continence in only 3 of 28 patients, but with repeated injections, 10/18 patients became fully continent, and 6/18 became almost continent. No significant gender-based differences were detected. Pre-augmentation BNIs were performed mainly for patients with slightly undersized bladders while on anticholinergic medication.

The sling procedure is popular for treating neurogenic sphincter insufficiency. However, we have encountered limitations with regard to young age. In our study, 8/14 patients became fully continent with a sling alone or combined with BNIs. Other studies have reported continence rates ranging from 14 to 88% with the sling operation (77%/43 [3], 88%/58 [7], 10/12 [14], 1/7 [15]). The effectiveness of BNI following a failed sling procedure has varied, with reported responses between 7 and 44% [3, 9, 13]. In our study, two patients received BNI after unsatisfactory outcomes of the sling procedure, one of which became fully continent. Nordhoof et al. reported a similar continence rate between bladder neck reconstruction and the sling procedure [3]. However, we did not perform bladder neck reconstructions in neurogenic bladders in this patient cohort.

BNC is rarely needed in patients with neurogenic bladder. However, 5/87 of our patients (all females) ended with BNC compared to 3.25% in the extensive series of Szymanski et al. [2]. Nowadays, we attempt to avoid BNCs to avoid possible adverse effects and enable easy removal of possible stones from the augmented bladder. Although BNC is very effective in treating incontinence in desperate cases, it may be complicated by stomal issues and stones [16].

Our study has limitations. It is a retrospective cohort spanning 30 years, making it impossible to assess the original urodynamic curves. Treatment protocols have changed over the years, including the availability of Botox treatments in our unit for the past 20 years. It is possible that some patients who underwent bladder augmentation in the 1990s may have avoided it if they were born later. The evolution of bladder neck procedures is also noteworthy as fewer patients now require repeated BNIs compared to the 1990s. Many patients in our study had only relative sphincter insufficiency, making it challenging to determine the necessity of bladder neck procedures. Moreover, there have been no fixed criteria for bladder neck procedure over the last 30 years. However, we have made an effort to describe the decision-making principle we employed. Our approach is prioritized treating the bladder first and considering bladder neck procedures only if the patient remained incontinent despite an adequate size and low-pressure bladder, or in conjunction with bladder augmentation when sphincter insufficiency was evident.

Not all patients adhered to the CIC program, and in some cases with borderline urodynamic findings, it was difficult to ascertain whether incontinence stemmed from poor sphincter function or inadequate implementation of the CIC program. In a few patients, rare instances of incontinence were associated solely with occasional bladder overfilling. Unfortunately, we were unable to measure leakage volumes. However, in the majority of treated cases, sphincter insufficiency was evident in urodynamic studies and detailed patient history. Furthermore, despite occasional minor incontinence, many patients expressed satisfaction and opted against pursuing additional major surgeries following bladder augmentation.

With our strategy to treat incontinence due to sphincter incompetency, partial or total continence was reached in 95% of patients. However, only 74% of our patients were fully continent, and 6% suffered from daily incontinence episodes. We conclude that the treatment of sphincter insufficiency after bladder augmentation is challenging in patients with neurogenic bladder.

## Abbreviations

CIC: Clean intermittent catheterization
BNP: Bladder neck procedure
BNI: Bladder neck injection
IQR: Interquartile range
BNC: Bladder neck closure
